# CG0009, a Novel Glycogen Synthase Kinase 3 Inhibitor, Induces Cell Death through Cyclin D1 Depletion in Breast Cancer Cells

**DOI:** 10.1371/journal.pone.0060383

**Published:** 2013-04-02

**Authors:** Hyun Mi Kim, Choung-Soo Kim, Je-Hwan Lee, Se Jin Jang, Jung Jin Hwang, Seonggu Ro, Jene Choi

**Affiliations:** 1 Department of Pathology, Asan Medical Center, University of Ulsan College of Medicine, Seoul, Korea; 2 Department of Urology, Asan Medical Center, University of Ulsan College of Medicine, Seoul, Korea; 3 Department of Hematology, Asan Medical Center, University of Ulsan College of Medicine, Seoul, Korea; 4 Asan Institute for Life Science, Asan Medical Center, Seoul, Korea; 5 Institute for Innovative Cancer Research, Asan Medical Center, Seoul, Korea; 6 CrystalGenomics Inc. Seoul, Korea; Broad Institute of Harvard and MIT, United States of America

## Abstract

Glycogen synthase kinase 3α/β (GSK3α/β) is a constitutively active serine/threonine kinase involved in multiple physiological processes, such as protein synthesis, stem cell maintenance and apoptosis, and acts as a key suppressor of the Wnt-β-catenin pathway. In the present study, we examined the therapeutic potential of a novel GSK3 inhibitor, CG0009, in the breast cancer cell lines, BT549, HS578T, MDA-MB-231, NCI/ADR-RES, T47D, MCF7 and MDA-MB-435, from the NCI-60 cancer cell line panel. Assessment of cytotoxicity, apoptosis and changes in estrogen-signaling proteins was performed using cell viability assays, Western blotting and quantitative real-time PCR. CG0009 enhanced the inactivating phosphorylation of GSK3α at Ser21 and GSK3β at Ser9 and simultaneously decreased activating phosphorylation of GSK3β at Tyr216, and induced caspase-dependent apoptosis independently of estrogen receptor α (ERα) expression status, which was not observed with the other GSK3 inhibitors examined, including SB216763, kenpaullone and LiCl. CG0009 treatment (1 µmol/L) completely ablated cyclin D1 expression in a time-dependent manner in all the cell lines examined, except T47D. CG0009 alone significantly activated p53, leading to relocation of p53 and Bax to the mitochondria. GSK3 inhibition by CG0009 led to slight upregulation of the β-catenin target genes, c-Jun and c-Myc, but not cyclin D1, indicating that CG0009-mediated cyclin D1 depletion overwhelms the pro-survival signal of β-catenin, resulting in cell death. Our findings suggest that the novel GSK3 inhibitor, CG0009, inhibits breast cancer cell growth through cyclin D1 depletion and p53 activation, and may thus offer an innovative therapeutic approach for breast cancers resistant to hormone-based therapy.

## Introduction

Glycogen synthase kinase 3 (GSK3) is a serine/threonine kinase expressed as two similar isoforms, α and β [1], [2]. GSK3 was initially identified as a metabolic regulator that phosphorylates and inhibits glycogen synthase [3]. GSK3 is a constitutively active enzyme in normal cells and undergoes rapid inhibition by stimuli [2], [4]. Activity of GSK3 is increased upon phosphorylation at Tyr216, whereas phosphorylation at Ser21 in GSK3α and Ser9 in GSK3β inhibits GSK3 activity [3], [5].

GSK3 is a key suppressor of the canonical Wnt signaling pathway of adenomatous polyposis coli (APC), axin and β-catenin, which is involved in embryonic cell fate determination and cell renewal [6], [7], [8]. GSK3β phosphorylates β-catenin, which leads to its destruction, thus suppressing signals that otherwise promote cell proliferation.

GSK3β inhibitors have been identified as therapeutic targets in Alzheimer’s disease, neurodegenerative disorders and bipolar disorder [9]. Recent studies have additionally shown that GSK3β inhibitors induce growth suppression and apoptosis in human chronic lymphocytic leukemia, glioma, colon cancer and renal cell carcinoma [10], [11], [12], [13]. Although GSK3β-promoted oncogenesis is a paradoxical issue, compelling evidence suggests that GSK3 is a target gene in malignancy. Firstly, GSK3β contributes to the promoter-specific recruitment of NF-kB [14], [15]. NF-kB DNA binding activity is reduced and its target gene products, including MMP-9, survivin, IAP-1, BCL-xL, TRAF1 and FLIP, are abrogated in GKS3β-null cells [16]. GSK3β inhibitors downregulate survivin and bcl-2 via inactivation of NF-kB and effectively kill leukemic cells [17]. Secondly, GSK3 promotes *MLL* oncogene-induced proliferation and transformation in *MLL* leukemia cell lines. GSK3 inhibitors reduce the proliferation of *MLL*-transformed myeloid progenitors, induce p27^Kip1^, a cyclin-dependent kinase inhibitor, and prolong the survival of a mouse model of *MLL*-associated leukemia [10]. Lastly, aberrant nuclear accumulation of GSK3β in renal cell carcinomas has been reported. GSK3 inhibitors suppress proliferation of renal cancer cells and exert a synergistic cytotoxic effect with docetaxel [13]. GSK3 inhibitors display diverse chemotypes, and most act in an ATP-competitive manner. Moreover, most GSK3 inhibitors do not discriminate between GSK3 isoforms α and β.

In the present study, we investigated the therapeutic efficacy of a novel GSK3 inhibitor, CG0009, in breast cancer cell lines of the NCI-60 panel. CG0009 induced inhibition of GSK3α/β, depletion of cyclin D1, activation of the p53-Bax pathway, and caspase-dependent apoptosis. These cytotoxic effects were not observed in kenpaullone-, SB216763- or LiCl-treated breast cancer cells. Our data support the utility of this compound as an effective therapeutic candidate for clinical development.

## Materials and Methods

### Cell Culture

Human breast cancer cell lines, BT549, HS578T, MDA-MB-231, NCI/ADR-RES, T47D, MCF7 and MDA-MB-435, which has been re-classified as the melanoma cell line M14 [18], and a colon cancer cell line, HCT116, were purchased from the National Cancer Institute (Bethesda, MD, USA). All cancer cell lines were cultured in RPMI-1640 (Invitrogen-Gibco, Carlsbad, CA, USA) supplemented with 10% FBS.

### GSK3 Inhibitors

CG0009 was provided by Crystal Genomics Inc. (Seoul, Korea). SB216763 was purchased from Enzo Life Sciences (Farmingdale, NY, USA). Kenpaullone and Lithium Chloride (LiCl) were obtained from Sigma-Aldrich (St. Louis, MO, USA).

### Cell Viability Assay and IC_50_ Values

IC_50_ values were determined using the CellTiter-Glo® Luminescent Cell viability Assay (Promega, Madison, WI, USA). Briefly, 3,000 cells per well were seeded in a 96-well plate. After 24 h, cells were treated with GSK3 inhibitors or DMSO in culture medium containing 5% FBS. After 48 h of treatment, cell viability was determined with the MicroLumatPlus LB96V Microplate Luminometer (Berthold Technologies GmbH & Co. KG, Bad Wildbad, Germany).

### Immunoprecipitation-Western Blot Analysis

MCF7 cells were seeded on 100 mm culture dishes and treated with 1 µmol/L CG0009 and lysed in 1% Chaps buffer (T&I, Bucheon, Korea). Cell lysates were incubated with Bax antibody (6A7; Santa Cruz Biotechnology) at 4°C overnight. Immunoprecipitates were captured using Protein A/G PLUS-Agarose beads (Santa Cruz Biotechnology) and separated by SDS-PAGE. Bax proteins were visualized with ECL solution (Thermo Fisher Scientific-Pierce, Rockford, IL, USA). Chemiluminescence of the membrane was detected using X-ray film (Agfa, Mortsel, Belgium).

### Western Blot Analysis

Whole-cell lysates were prepared in Cell Lysis Buffer (Cell Signaling Technology, Beverly, MA, USA). Proteins were separated by SDS-PAGE, and transferred to PVDF membranes using an iBlot™ dry blotting system (Invitrogen). Immunoblot analysis were done with the following primary antibodies: pGSK3α/β (Ser21/9), Caspase-9, PARP, pAKT (Ser473), AKT, pPI3K (Tyr458/Tyr199), PI3K, Cyclin D1, c-Jun, Aldolase, E-cadherin (Cell Signaling Technology), GSK3α/β, ERα, Bax (N-20), β-catenin, c-Myc, Prohibitin, N-cadherin, vimentin (Santa Cruz Biotechnology), p53, p21 (Upstate, Billerica, MA, USA), pGSK3β (Tyr216) (BD Biosciences, Franklin Lakes, NJ USA), Transferrin receptor (Zymed Laboratories, San Francisco, CA, USA), and β-actin (Sigma-Aldrich).

### AKT *in vitro* Kinase Assay

MCF7 cells were lysed with Cell Lysis Buffer (Cell Signaling Technology, 9803). One milligram of total cell extract was used per reaction. The K-LISA™ AKT Activity Kit (Calbiochem, Darmstadt, Germany, CBA019) was used with purified AKT (Calbiochem, 124006) as a positive control. Each experiment was repeated at least thrice.

### Quantitative Real-time Reverse Transcription-PCR (qRT-PCR)

Total cellular RNA was extracted using NucleoSpin® RNAII (Macherey-Nagel, Duren, Germany) and reverse-transcribed with SuperScript®II Reverse Transcriptase (Invitrogen). Gene expression levels were determined with the Bio-Rad iQ5 machine (Bio-Rad, Hercules, CA, USA) using SYBR Green (Invitrogen) with following primer sets: ERα, 5′-GGA GGG CAG GGG TGA-3′ (forward) and 5′-GGC CAG GCT GTT CTT CTT AG-3′ (reverse), yielding a 100 bp product, cyclin D1, 5′-CTA CTA CCG CCT CAC ACG CTT-3′ (forward) and 5′-GGC TTG ACT CCA GGG CT-3′ (reverse), yielding a 101 bp product, c-Jun, 5′-GTC CAC GGC CAA CAT GCT CA-3′ (forward) and 5′-TGT TTG CAA CTG CTG CGT TAG-3′ (reverse), yielding a 106 bp product, c-Myc, 5′-CAG CTG CTT AGA CGC TGG ATT-3′ (forward) and 5′-GTA GAA ATA CGG CTG CAC CGA-3′ (reverse), yielding a 131 bp product, GAPDH, 5′-GAA GGT GAA GGT CGG AGT C-3′ (forward) and 5′-GAA GAT GGT GAT GGG ATT TC-3′ (reverse), yielding a 226 bp product. The relative amount of target transcripts quantified using the standard curve method was normalized to the human GAPDH transcript level using Bio-Rad iQ5 2.0 Standard Edition Optical System Software V2.0.

### Transfection and Luciferase Assays

MCF7 and T47D cells were plated in 12-well plates and co-transfected with 0.5 µg of p53RE-containing reporter plasmid (p53-induced Luc; Stratagene- Agilent Technologies, Inc., Santa Clara, CA, USA) and 0.01 µg of *Renilla* luciferase plasmid (Promega), using Lipofectamine 2000 (Invitrogen) for MCF7 or the Neon™ Transfection System (Invitrogen) for T47D. At 24 h post-transfection, cells were treated with 1 µmol/L CG0009 or exposed to UV irradiation (20 J/m^2^). Cells were harvested after 24 h, and firefly luciferase activity measured in three independent experiments with the Dual-Luciferase® Reporter 1000 Assay System (Promega). Data were normalized to *Renilla* luciferase activity.

### Isolation of Mitochondria

Ten 150 mm dishes containing MCF7 cells were used for each treatment group. Cells were suspended on ice in MIB [mitochondria isolation buffer; 25 mmol/L Tris (pH 7.4), 250 mmol/L Sucrose, 1 mmol/L EDTA], homogenized using a 22-gauge needle syringe, and centrifuged at 1,000 *g* for 10 min, followed by 10,900 *g* for 10 min. The resulting pellets were resuspended in 1 mL of 50% Optiprep solution (Sigma-Aldrich) and layered into the bottom of an ultracentrifuge tube (R5; Hitachi Koki, Tokyo, Japan). Subsequently, 3 mL layers of 50%, 30% and 10% Optiprep solutions were sequentially added to the suspended solution. Following centrifugation in a Hitachi Himac CP80MX P80AT-0066 swinging bucket rotor instrument at 80,000 g for 3 h, mitochondrial fractions at the 10%/30% Optiprep interface were collected from the gradient, resuspended in MIB solution, and centrifuged at 3,000 g for 5 min. Supernatant fractions were recovered and re-centrifuged at 10,900 g for 10 min. Pellets were resuspended in MIB solution and stored.

## Results

### CG0009 Suppresses Breast Cancer Cell Proliferation *in vitro*

**Figure 1:**
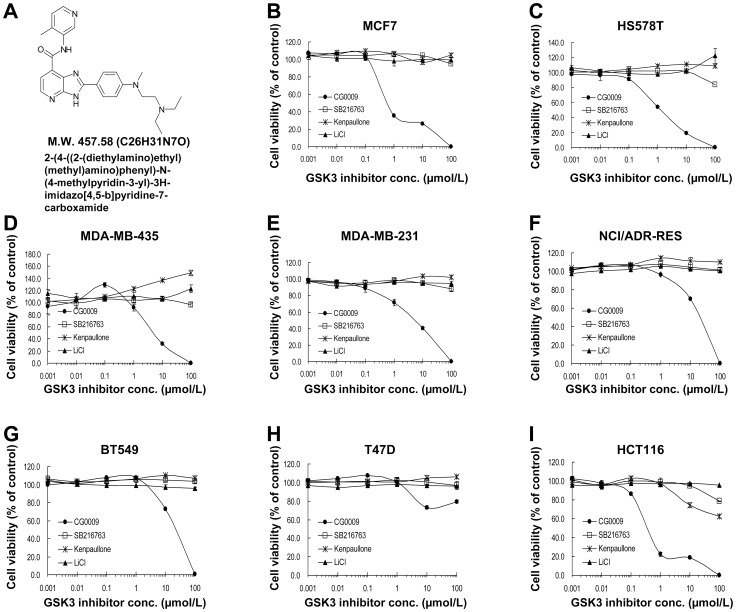
Anti-proliferative effects of GSK3 inhibitors on human cancer cell lines. (A) Structure of CG0009 {2-(4-((2-(diethylamino)ethyl)(methyl)amino)phenyl)-N-(4-methylpyridin-3-yl)-3H-imidazo[4,5-b]pyridine-7-carboxamide}. (B–I) Cells [MCF7 (B), HS578T (C), MDA-MB-435 (D), MDA-MB-231 (E), NCI/ADR-RES (F), BT549 (G), T47D (H), HCT116 (I)] were treated with 0.001, 0.01, 0.1, 1, 10 or 100 µmol/L of SB216763, kenpaullone, LiCl or CG0009 for 48 h, and incubated with CellTiter-Glo for 12 min. The percentage cell viabilities for each group were calculated by adjusting the DMSO control group to 100%. Points represent the mean values from three independent experiments; *bars* denote SD.

CG0009, a derivative of imidazopyridine carboxamide, was identified during extensive lead optimization of hits for inhibitors of GSK3 (Figure 1A). We initially evaluated the *in vitro* effect of CG0009, and compared it with those of other GSK3 inhibitors, SB216763, kenpaullone and lithium chloride (LiCl) [19]. CG0009 administered between concentrations of 0.001 and 100 µmol/L suppressed the growth of different breast cancer cell lines and the colon cancer cell line, HCT116, with a wide range of IC_50_ values except T47D cells (Table 1). The HCT116 cell line displayed the highest sensitivity to CG0009. In contrast, SB216763, kenpaullone and LiCl did not exert cytotoxic effects on all the cancer cell lines examined (Figure 1B–I). Based on these findings, CG0009-sensitive MCF7 and HCT116 and resistant T47D cell lines were selected for further study.

**Table 1:** IC_50_ of CG0009.

CellLine	CG0009	ERStatus	p53 Status
MCF7	0.49	+	Wild type
HS578T	1	−	D157E
MDA-MB-435	4.3	–	G266E
MDA-MB-231	4.9	–	R280K
NCI/ADR-RES	11	–	del 126–132
BT549	11	–	R249S
T47D	>100	+	L194F
HCT116	0.22	–	Wild type

(Unit : µmol/L).

### CG0009 Induces GSK3 Inactivation and Apoptosis in Breast Cancer Cells

**Figure 2:**
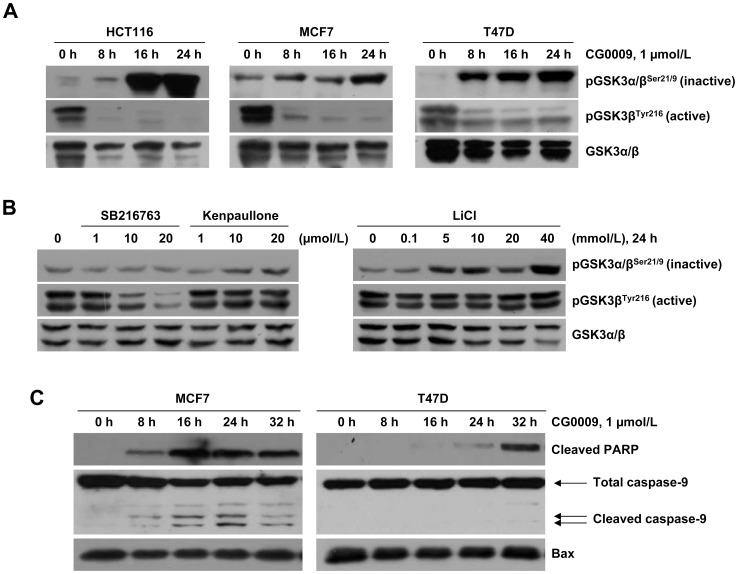
CG0009 inhibits GSK3 activity and induces caspase-dependent apoptosis. (A) HCT116, MCF7 and T47D cells were treated with 1 µmol/L of CG0009 for 8, 16 and 24 h. Expression levels of GSK3 phosphorylated at S21/9 (inactive form) and GSK3β phosphorylated at Y216 (active form) were analyzed using Western blotting. (B) MCF7 cells were treated with the indicated concentrations of SB216763, kenpaullone and LiCl for 24 h, and analyzed as described for (A). (C) MCF7 and T47D cells were exposed to 1 µmol/L CG0009, and analyzed by immunoblot analysis using caspase-9 and cleaved PARP-specific antibodies.

Next, we assessed GSK3 inhibition in HCT116, MCF7 and T47D cell lines after treatment with 1 µmol/L CG0009. The compound significantly enhanced phosphorylation of GSK3α Ser21 and GSK3β Ser9 (inactive forms of GSK3) and suppressed phosphorylation of GSK3β Tyr216 (active form of GSK3; the antibody recognized both isoforms), resulting in inhibition of GSK3 activity (Figure 2A). The level of total GSK3α/β was slightly decreased in the three cell lines. In contrast, kenpaullone and LiCl marginally induced inactive form of GSK3. SB216763 suppressed the active form of GSK3 at the highest dose (20 µmol/L), but did not induce the inactive GSK3 form (Figure 2B). CG0009 activated caspase-9 and PARP in sensitive MCF7 cells after 8 h treatment, but caused delayed PARP activation at 32 h and no detectable caspase-9 activation in T47D cells (Figure 2C). These results indicate that CG0009 is a potent inhibitor of GSK3α/β that promotes apoptosis in breast cancer cells.

### CG0009 Inhibits GSK3 in an AKT-independent Manner

**Figure 3:**
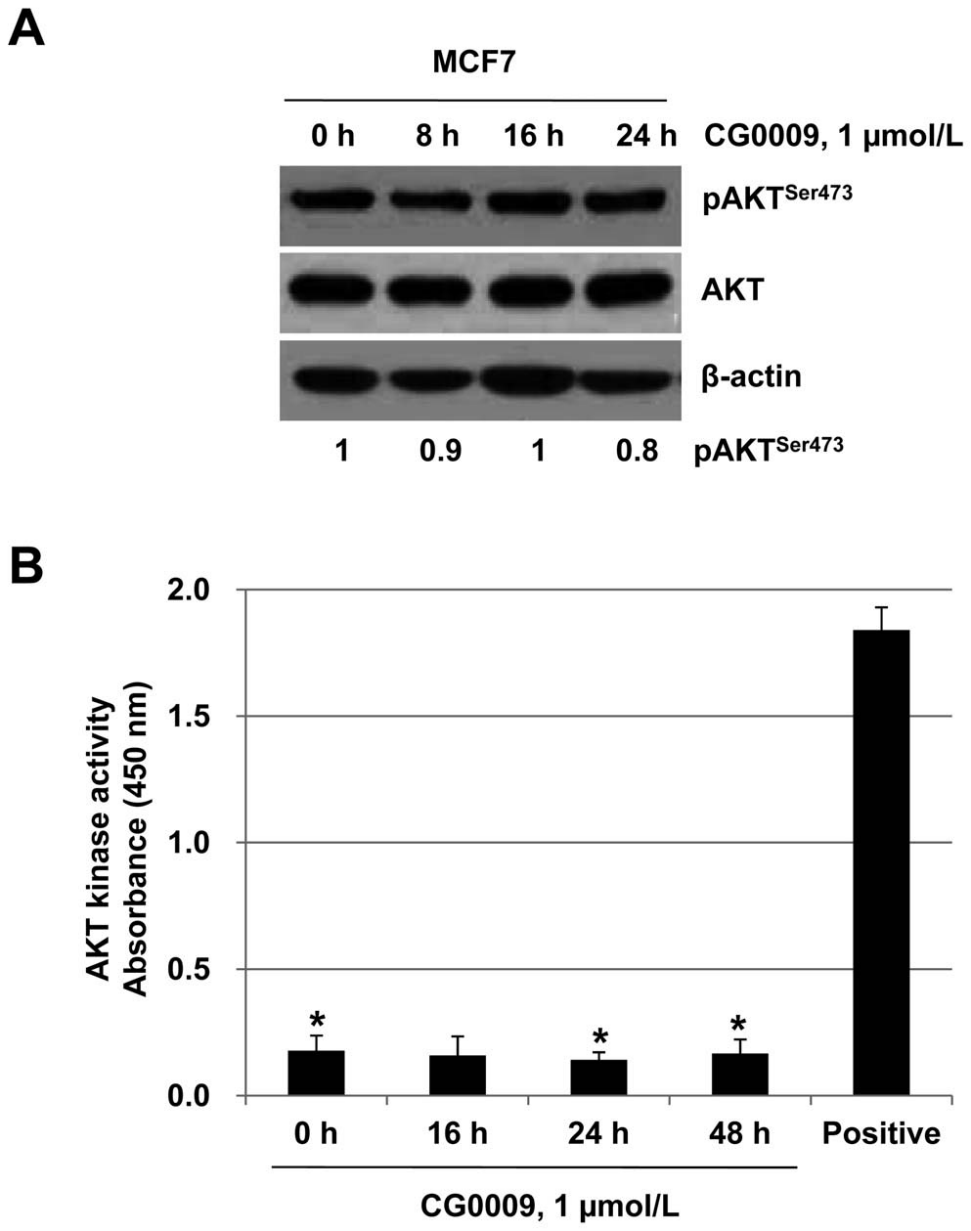
CG0009 does not change AKT activities. (A) MCF7 cells were treated with 1 µmol/L CG0009 and analyzed by immunoblotting with anti-pAKT^ser473^ and AKT antibodies. A densitometric analysis of pAKT^ser473^ was done with Quantity One software (BIO-RAD). (B) MCF7 cells were treated with 1 µmol/L CG0009 for the indicated times and whole cell lysates were prepared for *in vitro* AKT kinase assay. Purified recombinant AKT proteins were used as a positive control. Columns represent means of three independent experiments; *bars* denote SD (* *P*<0.05).

AKT phosphorylates GSK3, leading to its inactivation [20]. To test whether CG0009 inhibits GSK3 through AKT activation, we examined AKT phosphorylation in CG0009-treated MCF7 cells. The levels of pAKT on Ser473 fluctuated slightly during treatment up to 24 h, but these changes did not affect kinase activity (Figure 3A and B), indicating that CG0009 inhibits GSK3 independent of AKT.

### CG0009 Differentially Depletes ERα and Cyclin D1 in Sensitive and Resistant Breast Cancer Cells

**Figure 4:**
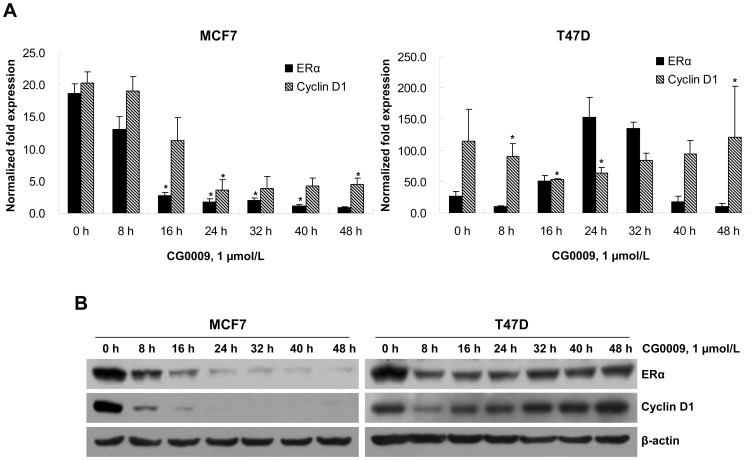
CG0009 suppresses ERα and cyclin D1 mRNA and protein expression. (A) MCF7 and T47D cells were treated with 1 µmol/L of CG0009 for up to 48 h, and the relative expression levels of ERα and cyclin D1 transcripts measured using qRT-PCR. Expression values were normalized to that of GAPDH. The mean value obtained from three independent experiments is shown in each column; *bar* denotes SD (*P<0.05 determined by *t* test). (B) Experiments were performed as described above, and total cell extracts analyzed by Western blotting using antibodies against ERα, cyclin D1, and β-actin as the loading control.

GSK3 protects estrogen receptor α (ERα) from proteasomal degradation and phosphorylates at Ser118 for full transcriptional activity of the receptor [21]. Accordingly, we examined the effects of CG0009 on estrogen signaling via qRT-PCR. Exposure to 1 µmol/L CG0009 significantly reduced the levels of ERα and its target cyclin D1 transcripts (∼ 5-fold) for up to 48 h in MCF7 cells (Figure 4A). In contrast, CG0009 caused oscillatory behavior in ERα and cyclin D1 expression patterns in T47D cells. ERα transcripts was initially reduced at 8 h, increased up to ∼ 8-fold at 24 h, and decreased again at 48 h. Cyclin D1 transcripts additionally showed a decrease and subsequent increase in levels. Consistent with qRT-PCR assay data, complete ablation of ERα and cyclin D1 proteins was observed in MCF7, but not T47D (Figure 4B). These results suggest that sensitivity to CG0009 is mediated through ERα and cyclin D1 proteins in MCF7 cells.

### CG0009 Downregulates Cyclin D1 in ERα-negative Breast Cancer Cells

**Figure 5:**
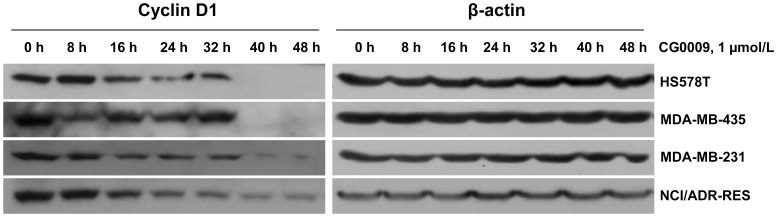
CG0009 downregulates cyclin D1 in ERα-negative cell lines. ERα-negative NCI/ADR-RES, MDA-MB-231, MDA-MB-435, HS578T and BT549 cells were treated with 1 µmol/L of CG0009 for up to 48 h. Subsequently, cells were harvested and 50 µg of total cellular proteins analyzed by immunoblotting with cyclin D1 antibodies. The β-actin level served as a loading control.

In view of the finding that CG0009 inhibits the proliferation of ERα-negative breast cancer cells, we further investigated its effect on the cyclin D1 protein level. Cells were treated with 1 µmol/L CG0009 for 8 to 48 h, and the cyclin D1 levels measured via Western blot analysis (Figure 5). Interestingly, CG0009 down-regulated cyclin D1 protein expression in ER-negative HS578T, MDA-MB-435, MDA-MB-231 and NCI/ADR-RES cells. Our data indicate that CG0009-mediated growth suppression and death are largely driven via cyclin D1 depletion in breast cancer cells, regardless of the ERα status.

### CG0009 Activates the p53-Bax Pathway

**Figure 6:**
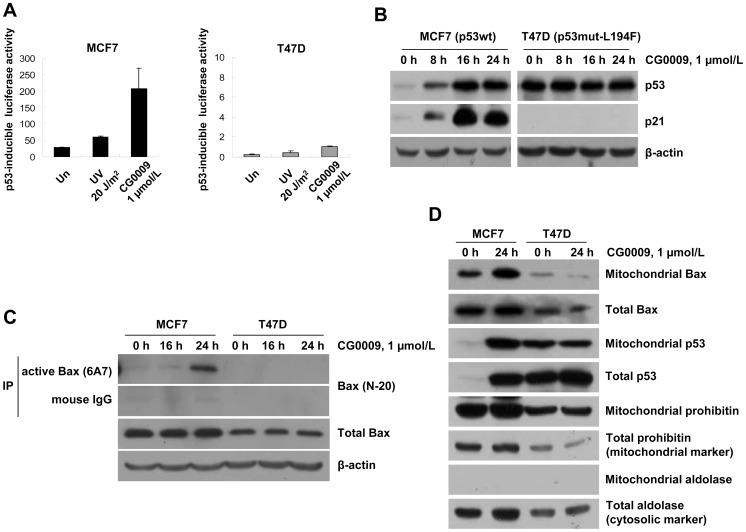
CG0009 activates the p53-Bax pathway and facilitates apoptosis in cells with wild-type p53. (A) MCF and T47D cells were transfected with a p53RE-containing reporter plasmid (p53-induced Luc), together with a *Renilla* luciferase vector for 24 h. Cells were exposed to UV (20 J/m^2^) or CG0009 (1 µmol/L) for 24 h. Luciferase activity was measured and normalized to control *Renilla* luciferase units. (B) MCF7 and T47D cells were treated with 1 µmol/L of CG0009 for the indicated times, and analyzed via Western blotting using p21 antibodies. (C) MCF7 and T47D cells were treated with CG0009 for the indicated times and extracted with buffer containing 1% CHAPS. Bax proteins were immunoprecipitated with anti-Bax (6A7) or mouse IgG antibodies, and analyzed by immunoblotting with anti-Bax antibodies. (D) After exposure to 1 µmol/L CG0009 for 24 h, MCF7 and T47D cells were fractionated into mitochondria, and analyzed with p53 and Bax-specific antibodies. Prohibitin is a mitochondrial marker and aldolase is a cytosolic marker.

GSK3β binds to p53 and facilitates p53-mediated transcriptional and apoptotic activity in the nucleus and mitochondria [12], [22]. Accordingly, we examined whether the p53 status determines the differential responses to CG0009 in MCF7 (containing wild-type p53) and T47D (with the L194F mutant of p53) cells. Initially, we tested p53-dependent transcription activity with the known p53-induced Luc construct after UV irradiation or CG0009 treatment. MCF7 cells displayed a dramatic increase in p53-mediated promoter activity 24 h after UV irradiation and CG0009 treatment. However, T47D cells subjected to either UV or CG0009 did not display promoter activation (the extent of increased promoter activity was less than 200-fold, compared with that observed in MCF7) (Figure 6A). Immunoblotting data revealed induction of p53 and p21 in MCF7, but not T47D, in response to CG0009, indicating that the latter cell line does not contain functional p53 (Figure 6B). Next, we examined whether p53 alters the effect of CG0009 in MCF7 and T47D cell lines. Specifically, the active form of Bax was immunoprecipitated with active conformation-specific 6A7 monoclonal Bax antibodies after CG0009 treatment, and active Bax protein levels determined using Western blot analysis. CG0009 significantly induced active Bax protein while the total level of Bax remained unchanged in MCF7 cells (Figure 6C). Moreover, active Bax and p53 were efficiently translocated to mitochondria. Notably, CG0009 did not affect the active Bax protein level or localization of Bax and p53 in T47D cells (Figure 6C and D). These findings indicate that integrity of the p53-Bax pathway is attributed to increased cell death in response to CG0009 in breast cancer cells.

**Figure 7:**
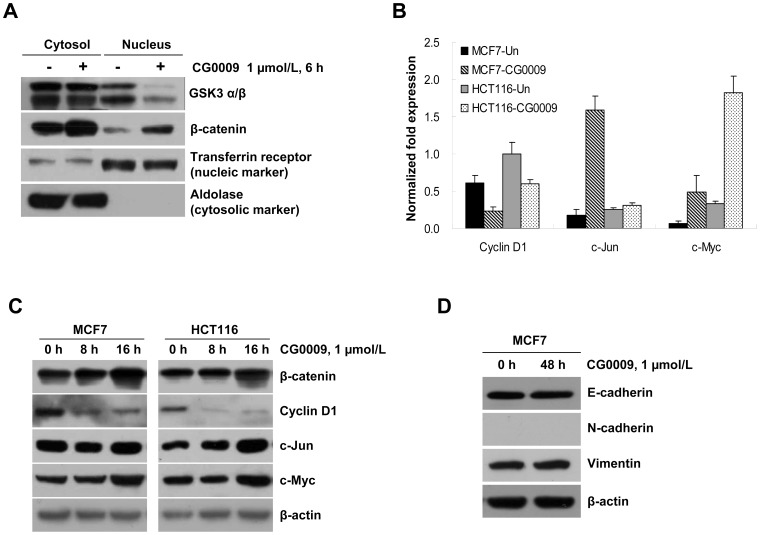
CG0009 activates β-catenin target gene expression to a minor extent. (A) MCF7 cells were treated with 1 µmol/L CG0009 for 6 h, fractionated into cytoplasm and nucleus, and probed with indicated antibodies. Transferrin receptor is a nuclear marker and aldolase is a cytosolic marker. (B) cyclin D, c-Jun and c-Myc mRNA transcripts were analyzed using qRT-PCR after CG0009 (1 µmol/L) treatment for 6 h in MCF7 and HCT116 cells. Data are presented as levels of cyclin D1, c-Jun, c-Myc transcripts relative to a control gene, GAPDH. The mean of three experiments is shown in each column; *bars* denote S.D. (C) HCT116 and MCF7 cells were treated with 1 µmol/L of CG0009 for the indicated times, and analyzed by Western blotting using β-catenin, cyclin D1, c-Jun and c-Myc antibodies. β-Actin was employed as the loading control. (D) MCF7 cells were treated with 1 µmol/L of CG0009 for 48 h. Whole cell lysates were prepared and analyzed by Western blotting using the indicated antibodies. Analysis of β-actin levels was used as a loading control.

CG0009 treatment causes slight activation of the Wnt-β-catenin pathway Inhibition of GSK3β may result in reduced phosphorylation of β-catenin, leading to its stabilization and accumulation in the nucleus, with subsequent activation of target genes [6]. In view of this theory, we analyzed whether CG0009 upregulates the expression of β-catenin target genes, such as c-Myc, c-Jun and cyclin D1, using the qRT-PCR assay. As shown in Figure 7A, incubation of MCF7 cells with 1 µmol/L CG0009 for 6 h resulted in increased β-catenin in the cytoplasm and nucleus. Furthermore, exposure to CG0009 induced transcription and protein expression of β-catenin target genes, such as c-Myc and c-Jun, in MCF7 and HCT116 cells. Interestingly, CG0009 significantly suppressed the mRNA level of cyclin D1, another well-known β-catenin target gene (Figure 7B and C). E-cadherin/β-catenin protein complexes play a major role in the epithelial-to-mesenchymal transition (EMT). An increase in CG009-mediated β-catenin may facilitate EMT in MCF7 cells. We examined whether CG009 changes the expression levels of mesenchymal proteins, vimentin and N-cadherin, or the epithelial protein E-cadherin. As shown in Figure 7D, CG0009 treatment did not induce EMT, as vimentin and N-cadherin did not increase, and E-cadherin did not decrease, after treatment. These data suggest that CG0009 activates the β-catenin pathway to a certain degree, but does not protect cells from apoptosis.

## Discussion

GSK3 inhibitors were initially identified as anti-diabetic targets, but more recent studies have highlighted these compounds as an emerging class of molecular therapeutic agents for human cancers. In this study, we have demonstrated for the first time that a novel imidazopyridine carboxamide-based GSK3 inhibitor, CG0009, inhibits proliferation, induces apoptosis cell death, and activates the p53-Bax pathway in breast cancer cells, predominantly via cyclin D1 depletion.

Our results showed that CG0009 induces significant growth inhibition and cell death in breast cancer cell lines, with a wide range of IC_50_ values (between 0.49 (MCF7) and 11 µmol/L (NCI/ADR-RES and BT549)), whereas other GSK3 inhibitors have little effect on cell growth at concentrations up to 100 µmol/L, which is much higher than that normally used for *in vitro* experiments, except LiCl (used at 10 ∼ 20 mmol/L, as shown in Figure 2B). Previous studies have reported that 10 µmol/L SB216763, 5 µmol/L kenpaullone, and 20 mmol/L LiCl markedly suppress growth in *MLL* leukemia cells, glioma cells and medullary thyroid cancer cells, respectively [10], [12], [23]. Rivest and colleagues additionally demonstrated that kenpaullone enhances prostate cancer cell proliferation at concentrations between 0.03 and 1 µmol/L and causes apoptosis at >1 µmol/L [24]. However, in our study, kenpaullone induced a slight increase in cell proliferation (Figure 1). Based on the findings, we conclude that breast cancer cells are generally less sensitive to GSK3 inhibitors, compared to other cancer cell types, and CG0009 may be a potent therapeutic agent specific for breast cancer. GSK3β inactivation through AKT hyperactivation was shown to increase anti-apoptotic Mcl-1 protein levels in breast cancer [25], [26]. Our results showed that CG0009 inhibits GSK3 independent of AKT (Figure 3).

CG0009 induced complete depletion of cyclin D1 in sensitive breast cancer cells (Figure 4 and 5). Note that the sensitivity to CG0009 and the time required for complete depletion of cyclin D1 is correlated. Most CG0009-hypersensitive MCF7 cells showed cyclin D1 depletion at 16 h, while the CG0009-moderately sensitive cell lines, HS578T, MAD-MB-435, and MDA-MB-231, exhibited depletion at 40 h following CG0009 treatment. Cyclin D1 depletion occurs earlier than actual cell growth inhibition and cell death in CG0009-moderately sensitive cell lines. This was evidenced by the fact that cell viability did not change at the same time and treatment dose that caused complete disappearance of cyclin D1.

These findings are inconsistent with previous data showing that phosphorylation of cyclin D1 at Thr286 by GSK3β enhances nuclear export and degradation [27]. Although cyclin D1 depletion by CG0009 may occur partly as a result of the decrease in ERα protein in MCF7 cells, we propose that this cyclin D1 decrease is a major cause of CG009-induced breast cancer cell death. Additionally, T47D cells expressed about 10-fold higher cyclin D1 transcripts, compared with the MCF7 cell line (Figure 4A). The *CCND1* gene encoding cyclin D1 at 11q13 is amplified in 30–40% of breast tumors, and its product is the rate-limiting factor for cell cycle progression [28], [29]. The 11q13.1–14.1 region is also amplified in T47D cells. Accordingly, we conclude that CG0009 is an effective therapeutic agent for breast cancer, regardless of functional ER status, and cyclin D1 overexpression is an unfavorable response marker for the agent.

In our experiments, p53 wild-type cells were more sensitive to CG0009, compared with p53 mutant cells. CG0009 activated p53, leading to a subsequent increase in p21, indicating that the drug activates stress-response pathways. GSK3β directly binds to p53, but its role is controversial at present. Watcharasit *et al* [30]. reported that GSK3β binds and promotes the transcriptional and apoptotic activities of p53 following camptothecin treatment, inconsistent with the finding that GSK3β suppresses p53-mediated apoptotic function after adriamycin, etoposide and 5-FU treatment in HCT 116 cells, and the GSK3 inhibitor, LY2119301, alone does not active stress responses [11]. In the current study, CG0009 alone activated p53-mediated transcription and facilitated the efficient translocation of Bax and p53 proteins to the mitochondria. Our results suggest that CG0009 is an efficient cytotoxic agent, which is sufficient for low-dose monotherapy, and p53 activation after GSK3 inhibition plays an important role in CG0009-induced breast cancer cell death.

Here, we have presented evidence that CG0009 implements cell death more efficiently than SB216763, kenpaullone and LiCl, through cyclin D1 ablation. While further studies are required to define the levels of normal physiologic and pathologic GSK3 activities, our preliminary data support the utility of CG0009 as an effective agent for the treatment of breast cancer, regardless of ERα expression status.

## References

[1] Woodgett JR (1990) Molecular cloning and expression of glycogen synthase kinase-3/factor A. EMBO J. 9: 2431–2438. 10.1002/j.1460-2075.1990.tb07419.x PMC552268 2164470

[2] Jope RS, Johnson GV (2004) The glamour and gloom of glycogen synthase kinase-3. Trends Biochem Sci 29: 95–102. 15102436 10.1016/j.tibs.2003.12.004

[3] Doble BW, Woodgett JR (2003) GSK-3: tricks of the trade for a multi-tasking kinase. J Cell Sci 116: 1175–1186. 12615961 10.1242/jcs.00384 PMC3006448

[4] Eldar-Finkelman H (2002) Glycogen synthase kinase 3: an emerging therapeutic target. Trends Mol Med 8: 126–132. 11879773 10.1016/s1471-4914(01)02266-3

[5] Cohen P, Goedert M (2004) GSK3 inhibitors: development and therapeutic potential. Nat Rev Drug Discov 3: 479–487. 15173837 10.1038/nrd1415

[6] van Noort M, Meeldijk J, van der Zee R, Destree O, Clevers H (2002) Wnt signaling controls the phosphorylation status of beta-catenin. J Biol Chem 277: 17901–17905. 11834740 10.1074/jbc.M111635200

[7] Sears R, Nuckolls F, Haura E, Taya Y, Tamai K, et al (2000) Multiple Ras-dependent phosphorylation pathways regulate Myc protein stability. Genes Dev 14: 2501–2514. 11018017 10.1101/gad.836800 PMC316970

[8] Cohen ED, Tian Y, Morrisey EE (2008) Wnt signaling: an essential regulator of cardiovascular differentiation, morphogenesis and progenitor self-renewal. Development 135: 789–798. 18263841 10.1242/dev.016865

[9] Jope RS, Yuskaitis CJ, Beurel E (2007) Glycogen synthase kinase-3 (GSK3): inflammation, diseases, and therapeutics. Neurochem Res 32: 577–595. 16944320 10.1007/s11064-006-9128-5 PMC1970866

[10] Wang Z, Smith KS, Murphy M, Piloto O, Somervaille TC, et al (2008) Glycogen synthase kinase 3 in *MLL* leukaemia maintenance and targeted therapy. Nature 455: 1205–1209. 18806775 10.1038/nature07284 PMC4084721

[11] Tan J, Zhuang L, Leong HS, Iyer NG, Liu ET, et al (2005) Pharmacologic modulation of glycogen synthase kinase-3beta promotes p53-dependent apoptosis through a direct Bax-mediated mitochondrial pathway in colorectal cancer cells. Cancer Res 65: 9012–9020. 16204075 10.1158/0008-5472.CAN-05-1226

[12] Kotliarova S, Pastorino S, Kovell LC, Kotliarov Y, Song H, et al (2008) Glycogen synthase kinase-3 inhibition induces glioma cell death through c-MYC, nuclear factor-kappaB, and glucose regulation. Cancer Res 68: 6643–6651. 18701488 10.1158/0008-5472.CAN-08-0850 PMC2585745

[13] Bilim V, Ougolkov A, Yuuki K, Naito S, Kawazoe H, et al (2009) Glycogen synthase kinase-3: a new therapeutic target in renal cell carcinoma. Br J Cancer 101: 2005–2014. 19920820 10.1038/sj.bjc.6605437 PMC2795437

[14] Ougolkov AV, Fernandez-Zapico ME, Savoy DN, Urrutia RA, Billadeau DD (2005) Glycogen synthase kinase-3beta participates in nuclear factor kappaB-mediated gene transcription and cell survival in pancreatic cancer cells. Cancer Res 65: 2076–2081. 15781615 10.1158/0008-5472.CAN-04-3642

[15] Steinbrecher KA, Wilson W 3rd, Cogswell PC, Baldwin AS (2005) Glycogen synthase kinase 3beta functions to specify gene-specific, NF-kappaB-dependent transcription. Mol Cell Biol 25: 8444–8455. 16166627 10.1128/MCB.25.19.8444-8455.2005 PMC1265740

[16] Hoeflich KP, Luo J, Rubie EA, Tsao MS, Jin O, et al (2000) Requirement for glycogen synthase kinase-3beta in cell survival and NF-kappaB activation. Nature 406: 86–90. 10894547 10.1038/35017574

[17] Ougolkov AV, Bone ND, Fernandez-Zapico ME, Kay NE, Billadeau DD (2007) Inhibition of glycogen synthase kinase-3 activity leads to epigenetic silencing of nuclear factor kappaB target genes and induction of apoptosis in chronic lymphocytic leukemia B cells. Blood 110: 735–742. 17463171 10.1182/blood-2006-12-060947 PMC1924475

[18] Rae JM, Creighton CJ, Meck JM, Haddad BR (2007) Johnson (2007) MDA-MB-435 cells are derived from M14 melanoma cells–a loss for breast cancer, but a boon for melanoma research. Breast Cancer Res Treat 104: 13–19. 17004106 10.1007/s10549-006-9392-8

[19] Holmes T, O’Brien TA, Knight R, Lindeman R, Symonds G, et al (2008) The role of glycogen synthase kinase-3beta in normal haematopoiesis, angiogenesis and leukaemia. Curr Med Chem 15: 1493–1499. 18537625 10.2174/092986708784638834

[20] Vivanco I, Sawyers CL (2002) The phosphatidylinositol 3-Kinase AKT pathway in human cancer. Nat. Rev. Cancer 2: 489–501. 12094235 10.1038/nrc839

[21] Grisouard J, Medunjanin S, Hermani A, Shukla A, Mayer D (2007) Glycogen synthase kinase-3 protects estrogen receptor alpha from proteasomal degradation and is required for full transcriptional activity of the receptor. Mol Endocrinol 21: 2427–2439. 17609434 10.1210/me.2007-0129

[22] Watcharasit P, Bijur GN, Song L, Zhu J, Chen X, et al (2003) Glycogen synthase kinase-3beta (GSK3beta) binds to and promotes the actions of p53. J Biol Chem 278: 48872–48879. 14523002 10.1074/jbc.M305870200 PMC1361697

[23] Kunnimalaiyaan M, Vaccaro AM, Ndiaye MA, Chen H (2007) Inactivation of glycogen synthase kinase-3beta, a downstream target of the raf-1 pathway, is associated with growth suppression in medullary thyroid cancer cells. Mol Cancer Ther 6: 1151–1158. 17363508 10.1158/1535-7163.MCT-06-0665

[24] Rivest P, Renaud M, Sanderson JT (2011) Proliferative and androgenic effects of indirubin derivatives in LNCaP human prostate cancer cells at sub-apoptotic concentrations. Chem Biol Interact 189: 177–185. 21111724 10.1016/j.cbi.2010.11.008

[25] Armanious H, Deschenes J, Gelebart P, Ghosh S, Mackey J, et al (2010) Clinical and biological significance of GSK-3β inactivation in breast cancer-an immunohistochemical study. Hum Pathol 41: 1657–1663. 20709358 10.1016/j.humpath.2010.04.015

[26] Ding Q, He X, Xia W, Hsu JM, Chen CT, et al (2007) Myeloid cell leukemia-1 inversely correlates with glycogen synthase kinase-3beta activity and associates with poor prognosis in human breast cancer. Cancer Res 67: 4564–4571. 17495324 10.1158/0008-5472.CAN-06-1788

[27] Diehl JA, Cheng M, Roussel MF, Sherr CJ (1998) Glycogen synthase kinase-3beta regulates cyclin D1 proteolysis and subcellular localization. Genes Dev 12: 3499–3511. 9832503 10.1101/gad.12.22.3499 PMC317244

[28] Johnson N, Speirs V, Curtin NJ, Hall AG (2008) A comparative study of genome-wide SNP, CGH microarray and protein expression analysis to explore genotypic and phenotypic mechanisms of acquired antiestrogen resistance in breast cancer. Breast Cancer Res Treat 111: 55–63. 17899364 10.1007/s10549-007-9758-6

[29] Kwek SS, Roy R, Zhou H, Climent J, Martinez-Climent JA, et al (2009) Co-amplified genes at 8p12 and 11q13 in breast tumors cooperate with two major pathways in oncogenesis. Oncogene 28: 1892–1903. 19330026 10.1038/onc.2009.34 PMC2722962

[30] Watcharasit P, Bijur GN, Zmijewski JW, Song L, Zmijewsk A, et al (2002) Direct, activating interaction between glycogen synthase kinase-3beta and p53 after DNA damage. Proc Natl Acad Sci U S A 99: 7951–7955. 12048243 10.1073/pnas.122062299 PMC123001

